# Six new species of dragon millipedes, genus *Desmoxytes* Chamberlin, 1923, mostly from caves in China (Diplopoda, Polydesmida, Paradoxosomatidae)

**DOI:** 10.3897/zookeys.577.7825

**Published:** 2016-04-05

**Authors:** Weixin Liu, Sergei Golovatch, Mingyi Tian

**Affiliations:** 1 Department of Entomology, College of Agriculture, South China Agricultural University, 483 Wushanlu, Guangzhou 510640, China; 2 Institute for Problems of Ecology and Evolution, Russian Academy of Sciences, Leninsky pr. 33, Moscow 119071, Russia; 3 Forschungsmuseum Alexander Koenig, Adenauerallee 160-162, Bonn 53113, Germany

**Keywords:** Desmoxytes, new species, troglobite, key, southern China

## Abstract

Six new species of *Desmoxytes* are described from southern China: *Desmoxytes laticollis* **sp. n.**, *Desmoxytes simplipoda* **sp. n.**, and *Desmoxytes similis* **sp. n.**, all three from caves in Guangdong Province; *Desmoxytes phasmoides* sp. n. also from a cave, and both epigean *Desmoxytes spiniterga* **sp. n.** and *Desmoxytes variabilis* **sp. n.**, the latter trio from Guangxi Zhuang Autonomous Region. A modified key to all 20 *Desmoxytes* species currently known to occur in China is given.

## Introduction

Millipedes in the genus *Desmoxytes* Chamberlin, 1923, belong to the tribe Orthomorphini, subfamily Paradoxosomatinae, family Paradoxosomatidae (Golovatch et al. 2012) and are often referred to as “dragon millipedes”. This genus is conspicuous in its species generally showing a dragon-like appearance, with strongly wing-, spine- or antler-shaped paraterga. *Desmoxytes* was first revised by Golovatch and Enghoff (1994), very recently reviewed by Likhitrakarn et al. (2015) to comprise 35 described species ranging from southeastern China south through Laos, Vietnam and Myanmar to southern Thailand and Western Malaysia. Only one species, *Desmoxytes planata* (Pocock, 1895), has attained a nearly pantropical distribution through commerce.

The dragon millipede fauna of China has also been summarized, keyed and shown to contain 14 species, including all 11 congeners hitherto known from caves (Golovatch et al. 2010, 2012, Liu et al. 2014, Golovatch 2015). Most of the cavernicolous *Desmoxytes* look highly troglomorphic and show spiniform paraterga.

The following 14 species are currently known to occur in mainland China, arranged in alphabetic order:

*Desmoxytes cornutus* Zhang & Li, 1982, from Guangxi, Guilin, Yangshuo.

*Desmoxytes draco* Cook & Loomis, 1924, from Jiangxi, Jiujiang, Lushan Mountain.

*Desmoxytes eupterygota* Golovatch, Li, Liu & Geoffroy, 2012, from two caves in Hunan, Chenzhou, Linwu.

*Desmoxytes getuhensis* Liu, Golovatch & Tian, 2014, from two caves in Guizhou, Ziyun, Getuhe National Geopark.

*Desmoxytes lingulata* Liu, Golovatch & Tian, 2014, from Guangxi, Guilin, Pingle, Chaotianyan.

*Desmoxytes longispina* Loksa, 1960, from a cave in Guangxi (an exact locality unknown).

*Desmoxytes lui* Golovatch, Li, Liu & Geoffroy, 2012, from a cave in Guangxi, Yongfu.

*Desmoxytes minutubercula* Zhang, 1986, from Guangxi, Tianlin.

*Desmoxytes nodulosa* Liu, Golovatch & Tian, 2014, from several caves in Guangxi, Hechi, Du’an.

*Desmoxytes parvula* Liu, Golovatch & Tian, 2014, from Guangxi, Du’an, Xia’ao.

*Desmoxytes planata* (Pocock, 1895), from a cave in Yunnan, Luxi, but actually nearly pantropical.

*Desmoxytes scolopendroides* Golovatch, Geoffroy & Mauriès, 2010, from a cave in Guangxi, Huanjiang and several caves in Du’an.

*Desmoxytes scutigeroides* Golovatch, Geoffroy & Mauriès, 2010, from several caves in Guangxi, Huanjiang and Du’an.

*Desmoxytes spinissima* Golovatch, Li, Liu & Geoffroy, 2012, from a cave in Guangxi, Fuchuan.

The present paper describes another six new species of *Desmoxytes* from southern China, including four presumed troglobites. Three of the new species are the first to be recorded in Guangdong Province, whereas a further three are from Guangxi Zhuang Autonomous Region which alone has already been known to support seven troglobitic species. Altogether, 20 species of *Desmoxytes* have now been recorded from China.

## Material and methods

The holotypes and a number of paratypes are deposited in the zoological collection of the South China Agricultural University, Guangzhou, China (SCAU), with some duplicates (paratypes) housed also in the Zoological Museum Alexander Koenig, Bonn, Germany (ZFMK), and the Zoological Museum, State University of Moscow, Russia (ZMUM).

Observations and dissections were performed using an Olympus SZ51 stereo microscope. The line drawings were prepared with the help of an Olympus SZX12 stereo microscope and a camera lucida attached to the scope. The photographs were taken with Canon EOS 40D and 7D cameras, further processed using Adobe Photoshop CS5 software.

The methods and terminology used here are after Golovatch et al. (2012).

## Taxonomic part

### Desmoxytes laticollis sp. n.

Taxon classificationAnimaliaPolydesmidaParadoxosomatidae

http://zoobank.org/9C11F333-5F13-4EBC-B111-2581CDB8D344

Figs 1A–B , 2 , 3

#### Holotype

♂ (SCAU), China, Guangdong, Qingyuan, Yingde Shi, Huanghua Xiang, Yanbei Cun, Cave Yangyan Dong, 24°18'32"N, 112°47'20"E, *ca* 450 m a.s.l., 2014-XII-30, leg. Mingyi Tian, Weixin Liu, Sunbin Huang & Xinhui Wang.

#### Paratypes.

13 ♂, 7 ♀ (SCAU), 1 ♂, 1 ♀ (ZMUM), 1 ♂, 1 ♀ (ZFMK), same locality and collecting data as the holotype.

#### Name.

To emphasize the collum being the broadest segment; adjective.

#### Diagnosis.

Keys out to the superficially most similar *Desmoxytes eupterygota* Golovatch, Li, Liu & Geoffroy, 2012 (Liu et al. 2014), especially so concerning metatergal ornamentation and a condensed solenophore, but differs by the collum being the broadest segment, as well as the femora, postfemora and tibiae conspicuously clavate in both sexes.

#### Description.

**Figure 1.**
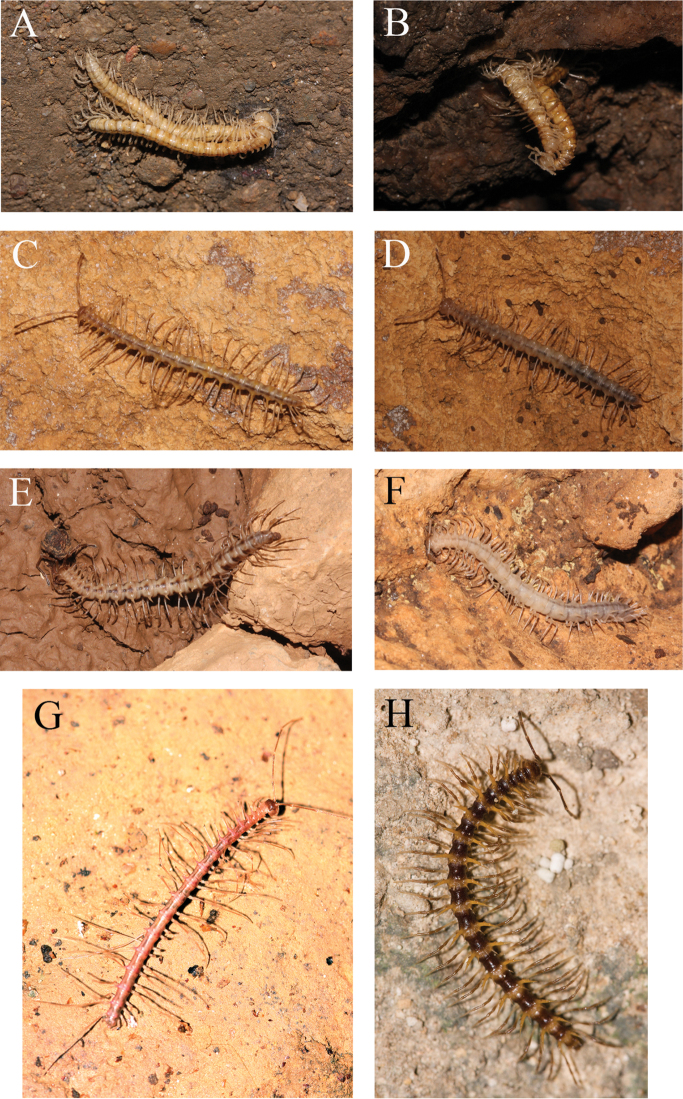
*In vivo* photographs. **A–B** a mating couple of *Desmoxytes laticollis* sp. n. **C–D** ♂ and ♀, *Desmoxytes simplipoda* sp. n. **E–F** 2 ♀, *Desmoxytes similis* sp. n. **G** ♂, *Desmoxytes phasmoides* sp. n. **H** ♂, *Desmoxytes variabilis* sp. n.

**Figure 2.**
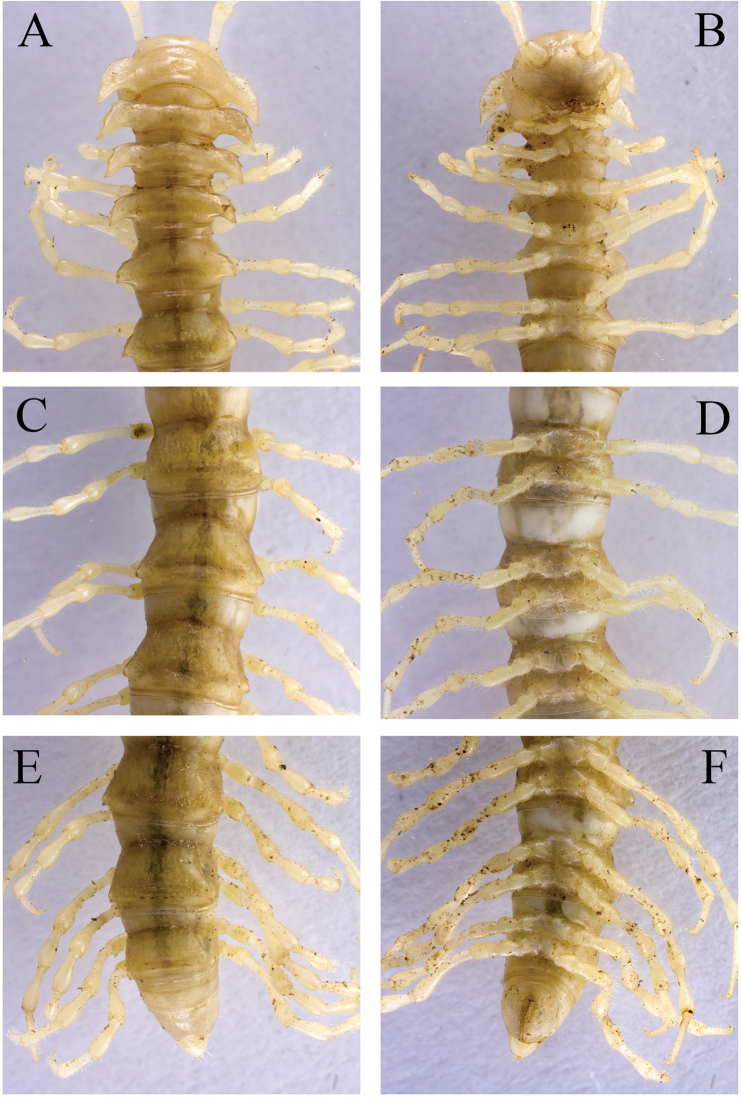
*Desmoxytes laticollis* sp. n., ♂ paratype from Cave Yangyan Dong. **A–B** anterior part of body, dorsal and ventral views, respectively **C–D** midbody segments, dorsal and ventral views, respectively **E–F** posterior part of body, dorsal and ventral views, respectively.

Length *ca* 26–27 mm (♂) or 28–29 mm (♀), width of midbody pro- and metazonae 2.0 and 2.5 (♂) or 2.2 and 2.5 mm (♀), respectively. Holotype 26 mm long, 2.0 and 2.5 mm wide on midbody pro- and metazonae, respectively, maximum width on collum 3.5 mm. In width, head < segment 8–16 < 5–7 < 4 < 3 < 2 < collum; starting with segment 17, body gradually tapering towards telson (Fig. 2). Live coloration rather uniformly yellowish to pallid (Fig. 1A–B). Head sparsely setose, epicranial suture distinct (Fig. 2A–B). Antennae long and slender, reaching back until posterior margin of segment 5 (♂) or middle of segment 4 (♀) when stretched dorsally; antennomeres 5 and 6 each with a compact apicodorsal group of bacilliform sensilla.

**Figure 3.**
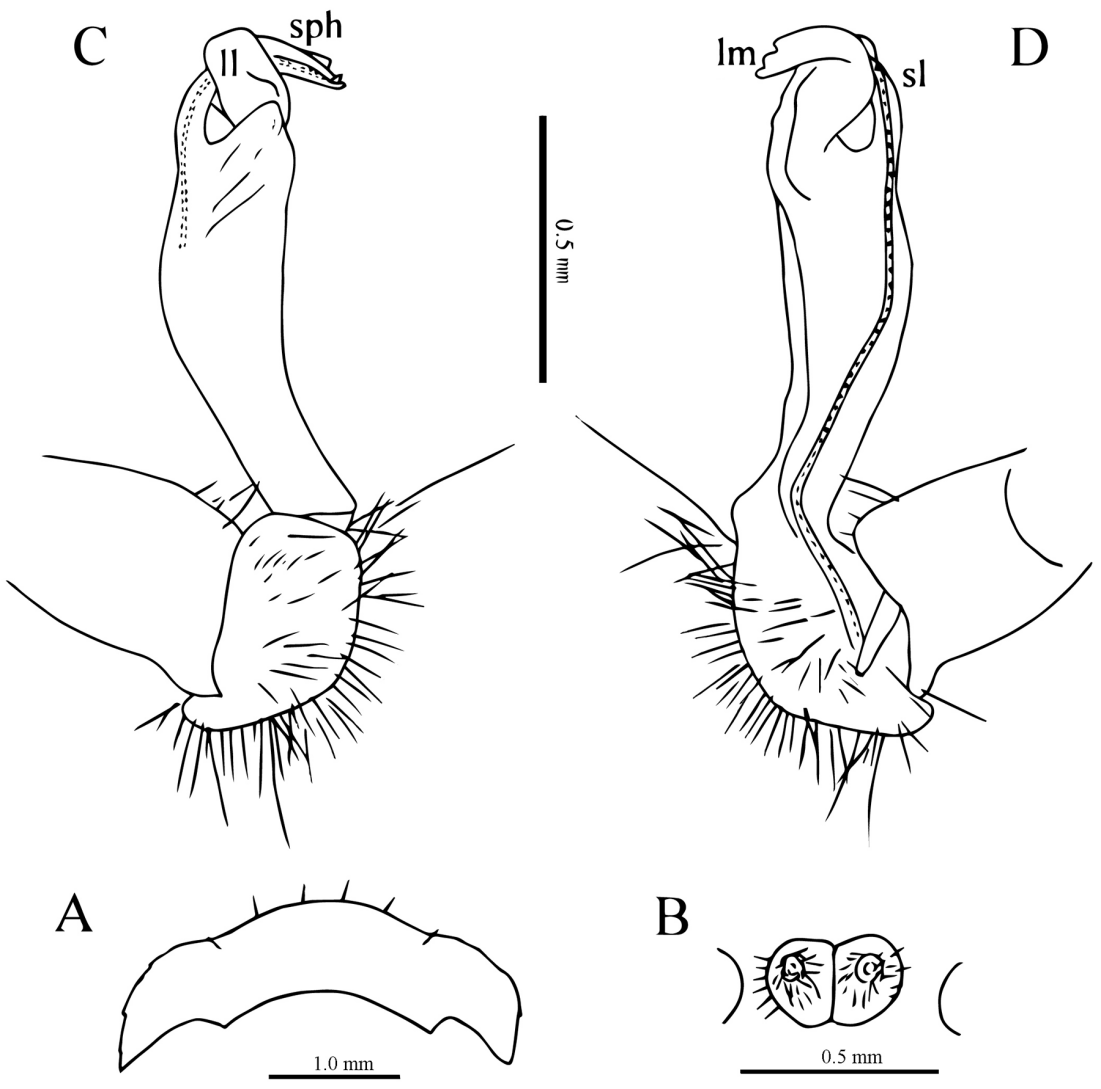
*Desmoxytes laticollis* sp. n., ♂ paratype from Cave Yangyan Dong. **A** collum, dorsal view **B** sternal processes between coxae 4, ventral view **C–D** right gonopod, lateral and mesal views, respectively.

Body with 20 segments. Tegument (Fig. 2) very strongly shining, prozonae faintly microalveolate; surface below paraterga of collum and those of segments 2–4 finely microgranulate. Collum (Figs 2A, 3A) with at least 2+2 setae at front margin, another 1+1 setae in the middle, hardly visible; paraterga on collum particularly strongly developed, wing-shaped, lying a little below a rather convex collum; paraterga on segments 2–4 clearly elevated above dorsum, thereafter rather poorly-developed, relatively small and crest-shaped, lying slightly below level of a faintly convex dorsum until segment 18, vestigial and lying far below level of a strongly convex dorsum on segment 19 (Fig. 2A, C, E); paraterga 1–4 with three especially strong denticles at lateral margin, two anterior of the denticles gradually disappearing towards segment 7, but caudalmost tooth persisting until segment 18 (Fig. 2A, C, E). Metaterga 2–4 each with 1+1 setae in anterior row; each of metaterga 5–19 additionally with 1+1 setae in posterior row, mostly poorly visible; paraterga a little more strongly developed in ♂ than in ♀, calluses very thin in poreless segments, slightly thicker in pore-bearing ones. Stricture between pro- and metazonae very narrow and deep. Ozopores entirely lateral, lying on top of caudal tooth on pore-bearing paraterga (Fig. 2A, C, E). Transverse sulcus evident and deep, smooth at bottom, reaching bases of paraterga on segments 7–16, incomplete (not reaching the bases of paraterga) in segments 5–6 and 17–18, vestigial in segment 19 (Fig. 2A, C, E). Epiproct subconical, clearly flattened dorsoventrally, mostly broad, subtruncate at a narrow apex, subapical lateral setae not borne on knobs (Fig. 2E–F). Hypoproct subtrapeziform, clearly emarginate at caudal margin, caudal setae distinctly separated (Fig. 2E–F). Pleurosternal carinae poorly-developed, only visible on segments 2 and 3 both in ♂ and ♀. Axial line missing.

Sterna sparsely setose, nearly flat, cross-impressions shallow, axial groove being especially superficial (Fig. 2B, D, F). A pair of paramedian, densely setose, low cones between ♂ coxae 4 (Figs 2B, 3B). Legs 1 short, following ones growing slightly, but increasingly long and slender towards telson, midbody legs *ca* 2.5 (♂) or 2.0 (♀) times as long as body height; femora, postfemora and tibiae conspicuously clavate distad, neither tarsal brushes nor adenostyles in ♂ and ♀ (Fig. 2).

Gonopods (Fig. 3C–D) suberect. Coxite short, subcylindrical, sparsely setose distodorsally, nearly 1/3 as long as telopodite. Prefemur densely setose, almost half as long as acropodite. Femorite long, slightly curved ventrad. Solenophore (**sph**) clearly condensed and divided into a large rectangular lobe on lateral side (= lamella lateralis, **ll**) and a distinct coiled part on mesal side (= lamella medialis, **lm**). Seminal groove running entirely on mesal side of femorite before entering onto a short, but evident and flagelliform solenomere (**sl**), the latter lying between **ll** and **lm**.

#### Remark.

Because of the pallid tegument and remarkably elongated antennae and legs, this species is most probably a troglobite.

### Desmoxytes simplipoda sp. n.

Taxon classificationAnimaliaPolydesmidaParadoxosomatidae

http://zoobank.org/AB383DC6-E76D-41D7-912F-6E75FC973DE0

Figs 1C–D , 4 , 5

#### Holotype

♂ (SCAU), China, Guangdong, Qingyuan, Yangshan Xian, Chengjia Xiang, Dabei Cun, Cave Kuangzhanyan, 24°46'28"N, 112°48'16"E, *ca* 140 m a.s.l., 2014-XII-28, leg. Mingyi Tian, Weixin Liu, Sunbin Huang & Xinhui Wang.

#### Paratypes.

1 ♂, 6 ♀ (SCAU), same locality and collecting data as the holotype.

#### Name.

To emphasize the legs being simple, devoid of modifications; adjective.

#### Diagnosis.

Using the latest key (Liu et al. 2014), this new species keys out to the superficially most similar *Desmoxytes longispina* (Loksa, 1960), especially so due to spiniform paraterga and a condensed solenophore, but differs by the legs being devoid of modifications.

#### Description.

All characters as in *Desmoxytes laticollis* sp. n., except as follows.

**Figure 4.**
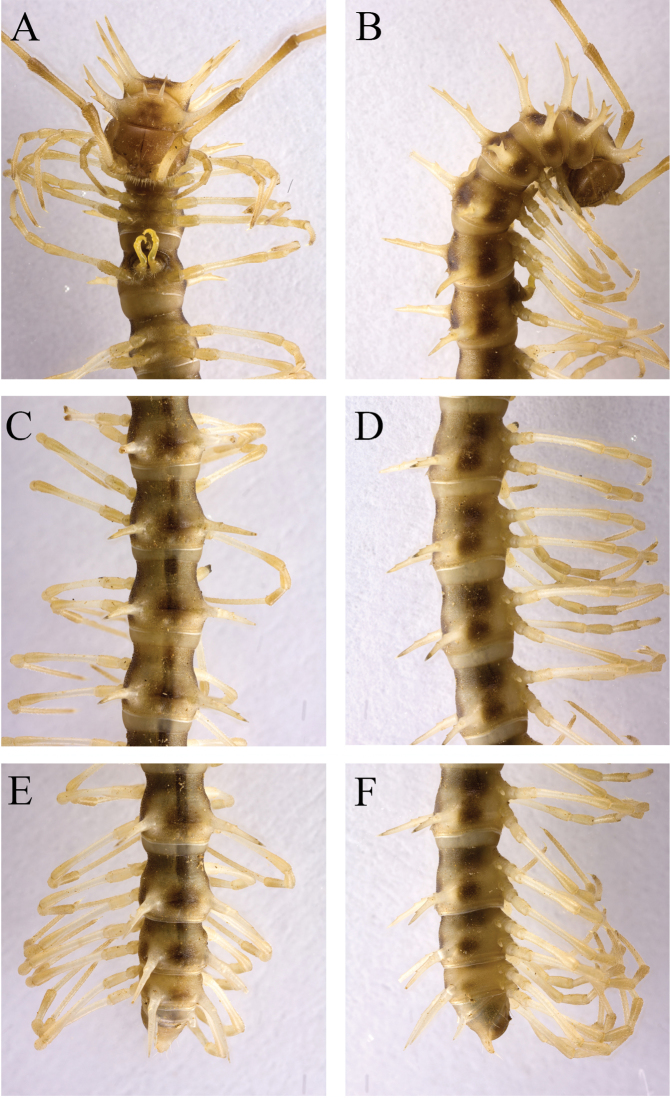
*Desmoxytes simplipoda* sp. n., ♂ holotype from Cave Kuangzhanyan. **A–B** anterior part of body, ventral and sublateral views, respectively **C–D** midbody segments, dorsal and lateral views, respectively **E–F** posterior part of body, dorsal and lateral views, respectively.

Length *ca* 28–29 mm (♂) or 31–33 mm (♀), width of midbody pro- and metazonae 1.8 and 4.0 (♂) or 2.5 and 4.5 mm (♀), respectively. Holotype 29 mm long, 1.8 and 4.0 mm wide on midbody pro- and metazonae, respectively. In width, head < segment 2–4 < collum < 5–16. Coloration (Fig. 1C–D) varying from dark brownish to nearly pallid, anterior part of body a little darker than posterior part. In holotype, head, as well as dorsal and both lateral sides of metaterga dark brownish; prozonae, paraterga, sterna, and legs pallid to yellowish (Fig. 4); apices of antennomeres 6 and 7 dark brownish (Fig. 1C–D). Antennae very long and slender, reaching back until posterior margin of segment 6 (♂) or segment 5 (♀) when stretched dorsally.

Prozonae very delicately microalveolate, metazonae rather finely shagreened and microgranulate (Fig. 4). Collum (Fig. 4A) with 3+3 evident setigerous spinules at fore margin, at least 1+1 much smaller spinules in the middle and 2+2 strongly enlarged spines (paramedian spines being larger than others) at posterior margin; following metaterga 2–18 showing a pattern of smaller 2+2 posterior spinules with invariably obliterated setae (paramedian two spinules a little larger, the other two located at base of each paratergum), while metaterga 19 with 1+1 posterior spinules (Fig. 4C, E). Paraterga (Fig. 4) very strongly developed, spiniform, on collum with four evident anteromarginal denticles; all following paraterga long, straight, also spiniform, about as high as metatergal height in ♂, a little shorter in ♀; paraterga 2–18 with 2–3 evident denticles frontally. Paraterga 2–9 directed more dorsad than laterad, nearly erect above dorsum; following paraterga directed a little caudad, but ending up clearly above dorsum. Ozopores conspicuous, located a little above first denticle from lateral side of pore-bearing paraterga (Fig. 4B–F). Transverse sulcus present on segments 2–19, but complete and reaching bases of paraterga only on segments 6–15 (Fig. 4C, E).

**Figure 5.**
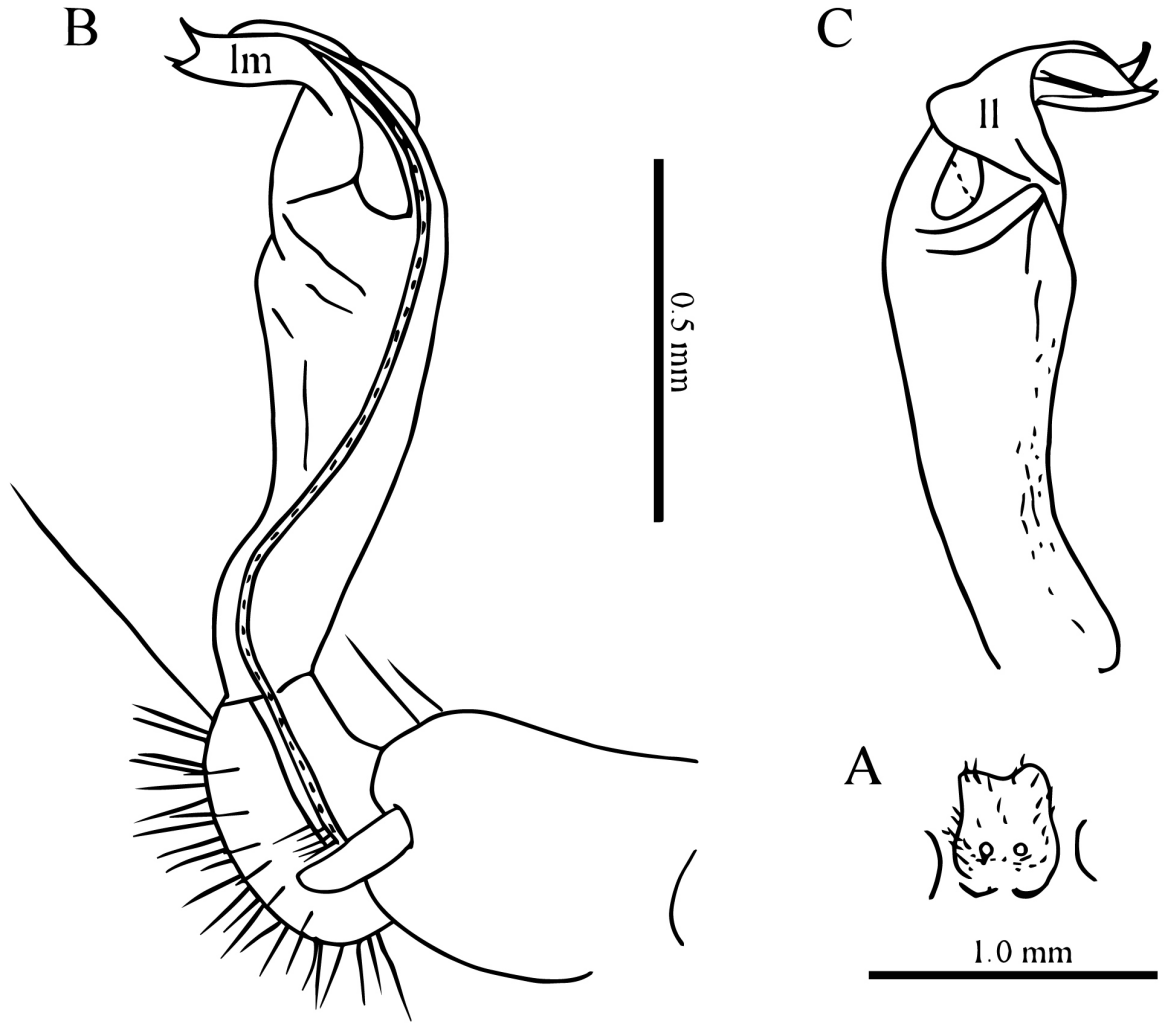
*Desmoxytes simplipoda* sp. n., ♂ holotype from Cave Kuangzhanyan. **A** sternal process between coxae 4, ventral view **B–C** entire right gonopod and its distal half, mesal and lateral views, respectively.

Sterna sparsely setose, cross-impressions evident. A large, median, sparsely setose process with two small pores at base between ♂ coxae 4 (Figs 4B, 5A). Legs devoid of modifications (Fig. 4A–B), *ca* 2.8–3.0 (♂) or 2.5 (♀) times as long as midbody height.

Gonopods (Fig. 5B–C) simple. Coxite stout, about 1/3 as long as telopodite. Prefemur short, less than half the length of acropodite. Femorite long, suberect. Solenophore strongly condensed and divided into a large subtriangular lamella lateralis (**ll**) and a terminally evidently bifid lamella medialis (**lm**).

#### Remark.

Judging by the extremely elongated antennae and legs, this species seems to be a troglobite.

### Desmoxytes similis sp. n.

Taxon classificationAnimaliaPolydesmidaParadoxosomatidae

http://zoobank.org/3E252D45-5DE5-4F32-B3A6-9F855E84A437

Figs 1E–F , 6 , 7

#### Holotype

♂ (SCAU), China, Guangdong, Qingyuan, Yingde Shi, Qingkeng Zhen, Bangjiao Cun, Cave Bangjiao Dong, 24°25'09"N, 112°57'16"E, *ca* 230 m a.s.l., 2014-XII-29, leg. Mingyi Tian, Weixin Liu, Sunbin Huang & Xinhui Wang.

#### Paratypes.

1 ♂, 4 ♀ (SCAU), 1 ♂, 1 ♀ (ZFMK), same locality and collecting data as the holotype.

#### Name.

To emphasize the particular similarities to *Desmoxytes simplipoda* sp. n.; adjective.

#### Diagnosis.

This species seems to be especially similar to *Desmoxytes simplipoda* sp. n., from Yangshan, Qingyuan, Guangdong, but differs by the setose process between ♂ coxae 4 showing a large pore and the lamella medialis of the gonopod solenophore a small lobule at about midlength.

#### Description.

All characters as in *Desmoxytes simplipoda* sp. n., except as follows.

**Figure 6.**
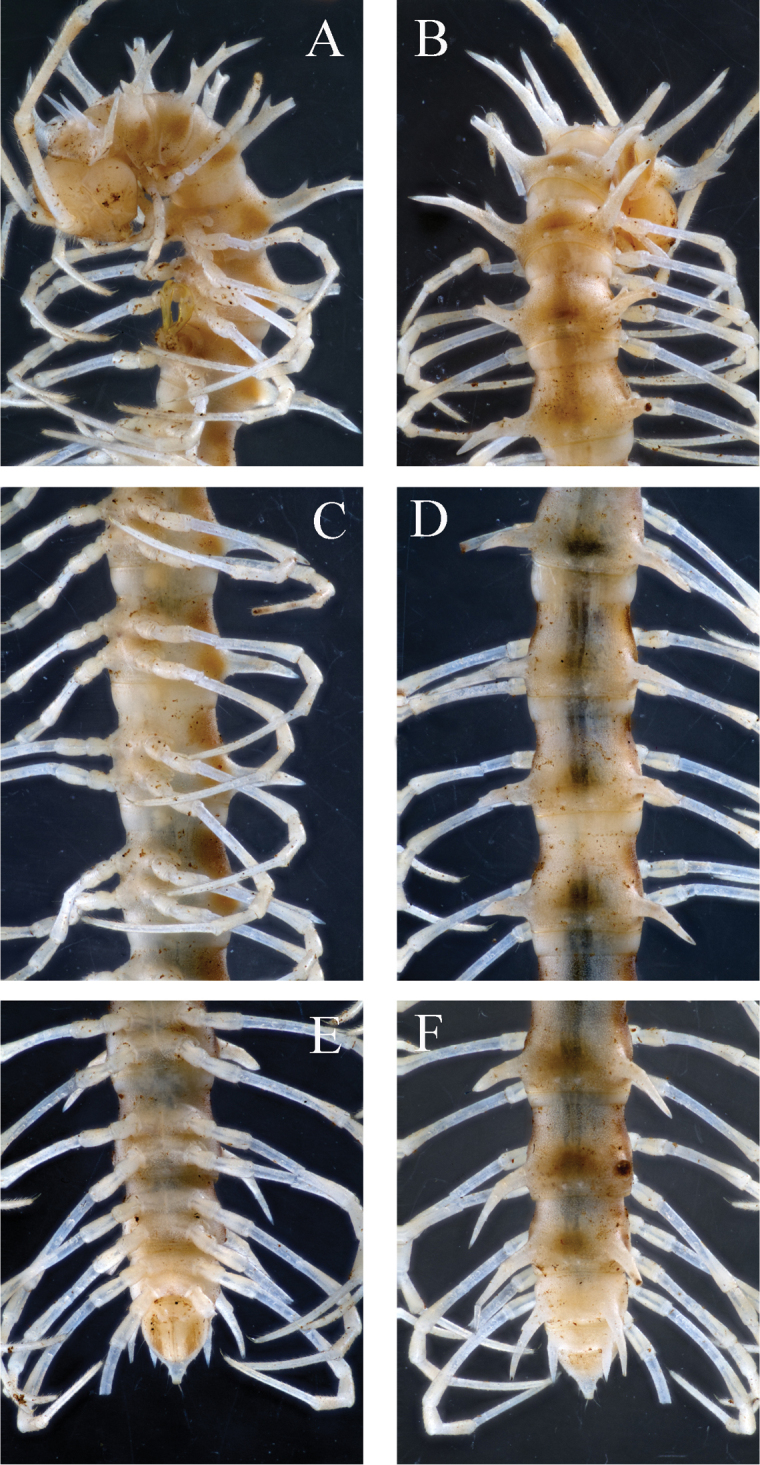
*Desmoxytes similis* sp. n., ♂ holotype from Cave Bangjiao Dong. **A–B** anterior part of body, subventral and dorsal views, respectively **C–D** midbody segments, subventral and dorsal views, respectively **E–F** posterior part of body, ventral and dorsal views, respectively.

Length *ca* 25–26 mm (♂) or 27–29 mm (♀), width of midbody pro- and metazonae 1.5 and 4.0 (♂) or 2.5 and 4.5 mm (♀), respectively. Holotype 26 mm long, 1.5 and 4.0 mm wide on midbody pro- and metazonae, respectively. In width, head < collum < segment 2–8 < 9–16. Coloration rather uniformly light brownish to pallid (Figs 1E–F, 6).

Paraterga of collum (Fig. 6A–B) directed laterad; following paraterga 2–18 directed dorsad and a little caudad; paraterga 19 directed caudad, but all paraterga ending up clearly much above dorsum (Fig. 6). Transverse sulcus very vague, only traceable in segments 3–18 (Fig. 6B, D, F).

**Figure 7.**
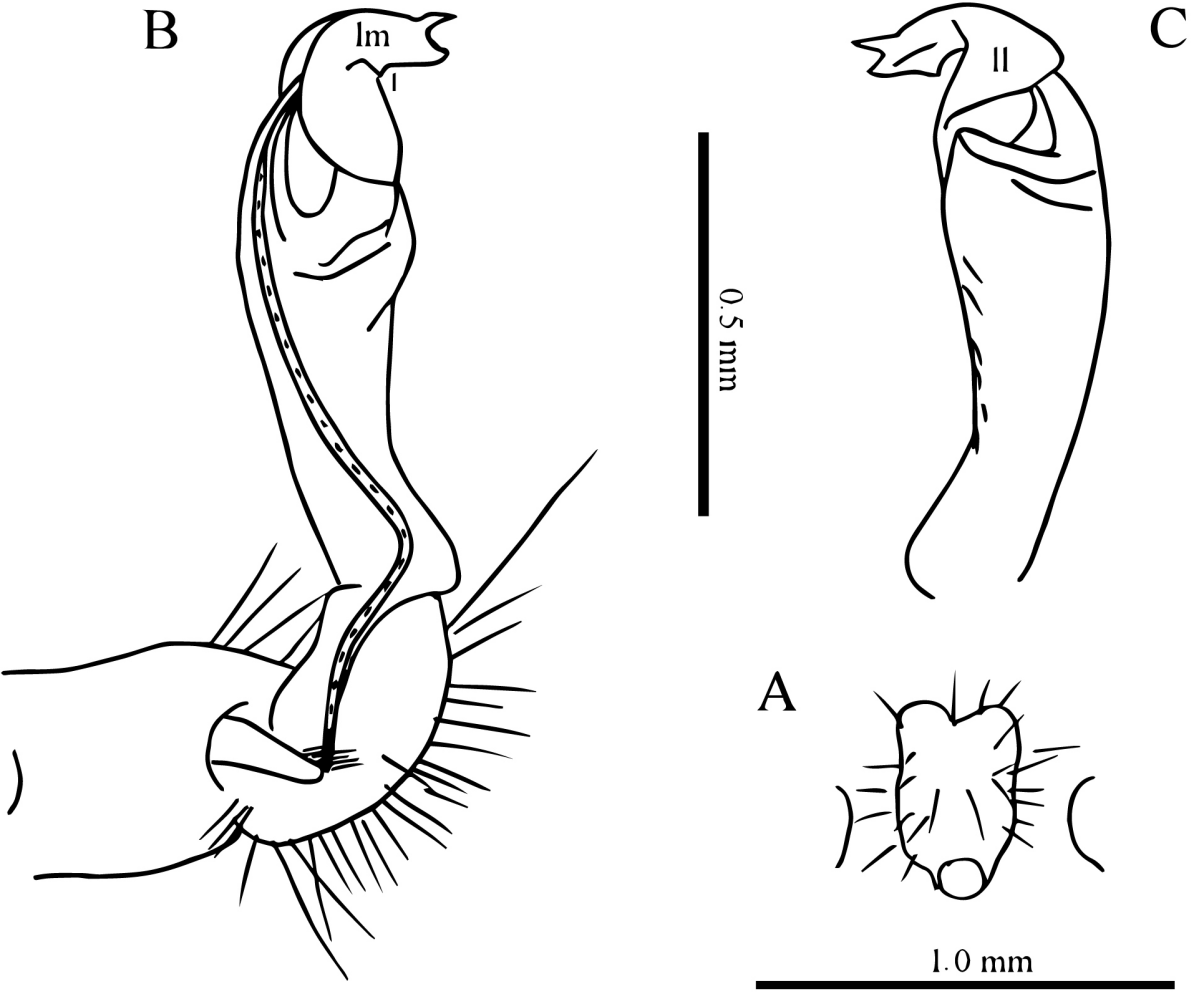
*Desmoxytes similis* sp. n., ♂ paratype from Cave Bangjiao Dong. **A** sternal process between coxae 4, ventral view **B–C** entire left gonopod and its distal half, mesal and lateral views, respectively.

A large, median, setose process with a large central pore at bottom between ♂ coxae 4 (Fig. 7A). Legs devoid of modifications, about 2.5 (♂) or 2.0 (♀) times as long as midbody height.

Gonopods (Fig. 7B–C) short. Coxite less than 1/3 the length of telopodite. Prefemur about half as long as acropodite. Femorite very short, slightly enlarged distad. Solenophore strongly condensed and divided into a large subtriangular lamella lateralis (**ll**) and a terminally evidently bifid lamella medialis (**lm**), the latter with a very small lobule (**l**) at about midlength.

#### Remarks.

This species seems to be very similar to *Desmoxytes simplipoda* sp. n., from Yangshan, Qingyuan, Guangdong. The only differences are outlined in the above diagnosis. Because of the nearly pallid tegument and extremely elongated antennae and legs, this species seems to be a troglobite.

### Desmoxytes phasmoides sp. n.

Taxon classificationAnimaliaPolydesmidaParadoxosomatidae

http://zoobank.org/258D7BBE-ECB2-4123-A03A-390B70438280

Figs 1G , 8 , 9

#### Holotype

♂ (SCAU), China, Guangxi, Baise, Lingyun Xian, Jiayou Zhen, Yangli Cun, Cave Fengliu Dong, 2015-VI-9, leg. Mingyi Tian, Weixin Liu, Xinhui Wang & Mingruo Tang.

#### Paratypes.

1 ♂, 1 ♀ (SCAU), 1 ♂ (ZFMK), same locality and collecting data as the holotype.

#### Name.

To emphasize that superficially this new species somewhat resembles a stick insect, Phasmatodea; noun in apposition.

#### Diagnosis.

Keys out to the superficially most similar *Desmoxytes minutubercula* Zhang, 1986 (Liu et al. 2014), especially so due to long spiniform paraterga and a particularly condensed solenophore, but differs by a pair of rounded, setose processes present between ♂ coxae 4 and the gonopod lamella medialis showing a distinct spine.

#### Description.

All characters as in *Desmoxytes laticollis* sp. n., except as follows.

Length of both sexes *ca* 27–29 mm, width of midbody pro- and metazonae 1.3–1.5 and 2.8–3.0 mm, respectively. Holotype 29 mm long, 1.5 and 3.0 mm wide on midbody pro- and metazonae, respectively. In width, segment 2–4 < collum < head < 5–7 < 8–18. Coloration rather uniformly light brownish, some metaterga and bases of paraterga pinkish (Fig. 1G). Antennae very long and slender, reaching back until posterior margin of segment 8 (7) (♂) or 6 (♀) when stretched dorsally.

**Figure 8.**
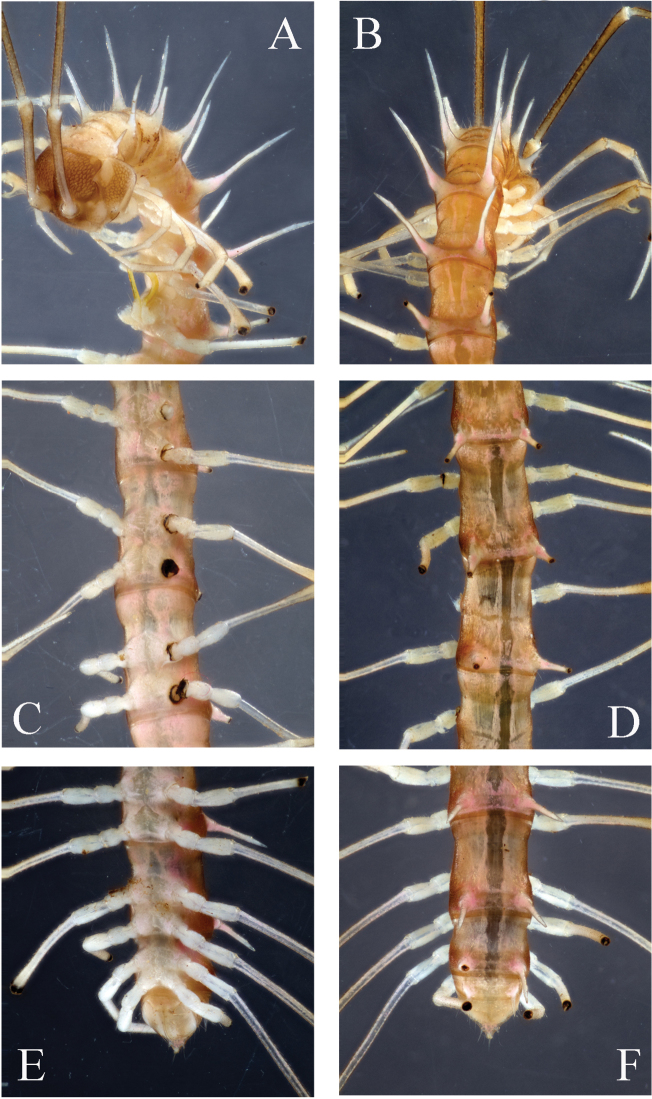
*Desmoxytes phasmoides* sp. n., ♂ paratype from Cave Fengliu Dong. **A–B** anterior part of body, subventral and dorsal views, respectively **C–D** midbody segments, ventral and dorsal views, respectively **E–F** posterior part of body, ventral and dorsal views, respectively.

**Figure 9.**
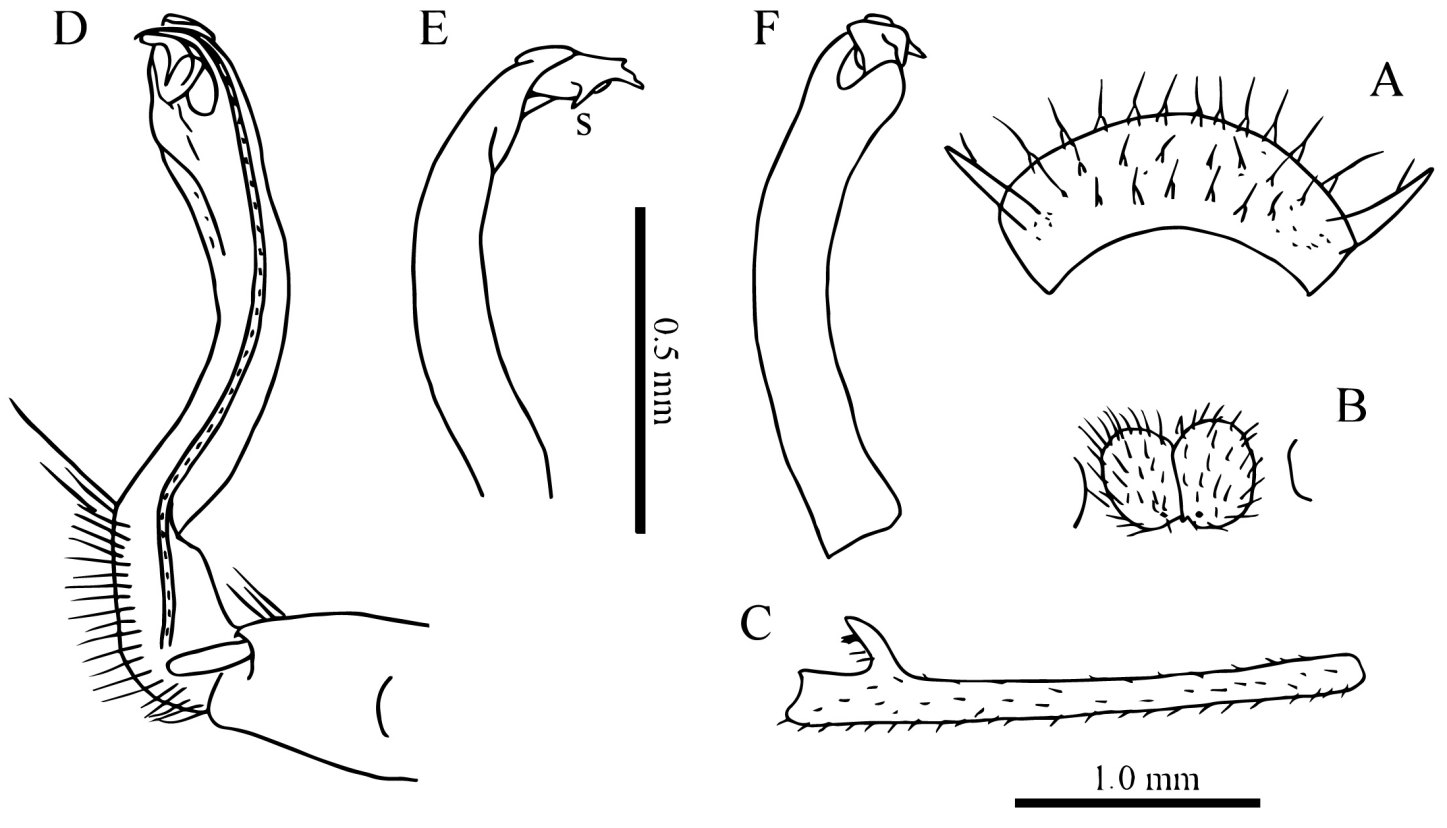
*Desmoxytes phasmoides* sp. n., ♂ paratype from Cave Fengliu Dong. **A** collum, dorsal view **B** sternal processes between coxae 4, ventral view **C** femur 6, front view **D–F** entire right gonopod and its distal half, mesal, ventral and lateral views, respectively.

Tegument shining and smooth, prozonae faintly microalveolate; metazonae finely microgranulate (Fig. 8). Collum (Fig. 8A) with at least 6+6 anterior, 4+4 (5) intermediate and 3+3 posterior setigerous spines; paraterga on collum spiniform, directed dorsolaterad, with a setigeous spine anteriorly at distal 1/3 (Figs 8A, 9A). Metaterga 2–4 each with 4+4 anterior, 3+3 intermediate and 5+5 posterior setigerous tubercles; metaterga 5–19 with a pattern of 5+5 setigerous tubercles anteriorly, these occasionally arranged in two transverse rows, as well as 4+4 between paraterga and at least 5+5 setigerous tubercles at posterior margin. Paraterga 2–18 (Fig. 8) extremely long, straight, spiniform, simple, usually with 2–3 very small setigerous denticles on lateral side; only paraterga 19 directed caudad. Ozopores inconspicuous, lying at base of pore-bearing paraterga on lateral side (Fig. 8D). Transverse sulcus incomplete, present on segments 6–18 (Fig. 8B, D, E).

Sterna modestly setose, cross-impressions very shallow (Fig. 8C, E). A pair of paramedian, rounded, setose processes between ♂ coxae 4 (Fig. 9B). Legs long and slender, midbody legs *ca* 4.5 (♂) or 3.5 (♀) times as long as body height; ♂ femur 6 with a very evident apophysis at distal 1/4 (Fig. 9C).

Gonopods (Fig. 9D–F) subfalcate. Coxite very short, less than 1/3 as long as telopodite. Prefemur short, less than half as long as acropodite. Femorite rather long, curved ventrad. Solenophore highly condensed, divided into a small, rectangular lamella lateralis and a simple lamella medialis, the latter with a distinct spine (**s**) at about midlength; solenomere very short and flagelliform.

#### Remark.

Because of the pallid tegument and extremely elongated antennae and legs, this species may well be a troglobite.

### Desmoxytes spiniterga sp. n.

Taxon classificationAnimaliaPolydesmidaParadoxosomatidae

http://zoobank.org/9D8F7BAF-4AB7-43F4-BD54-2B5A8F1925EA

Figs 10 , 11

#### Holotype

♂ (SCAU), China, Guangxi, Hechi, Huanjiang Xian, near Cave Gui Dong II, Secondary forest, litter, Berlese extraction after sifting, 2007-V-18, leg. Louis Deharveng & Anne Bedos (CHIgx07-18-17).

#### Paratype.

1 ♂ (SCAU), same locality and collecting data as the holotype.

#### Name.

To emphasize the metaterga showing very evident, spiniform, setigerous paraterga; adjective.

#### Diagnosis.

Keys out to the superficially most similar *Desmoxytes draco* Cook & Loomis, 1924 (Liu et al. 2014), judging from the ornamentation of metaterga, but differs by legs showing no modifications, in the metaterga supplied with more numerous setigerous spines, as well as the rather short femorite of the gonopod and the clearly coiled solenophore (cf. Kraus 2012).

#### Description.

All characters as in *Desmoxytes laticollis* sp. n., except as follows.

**Figure 10.**
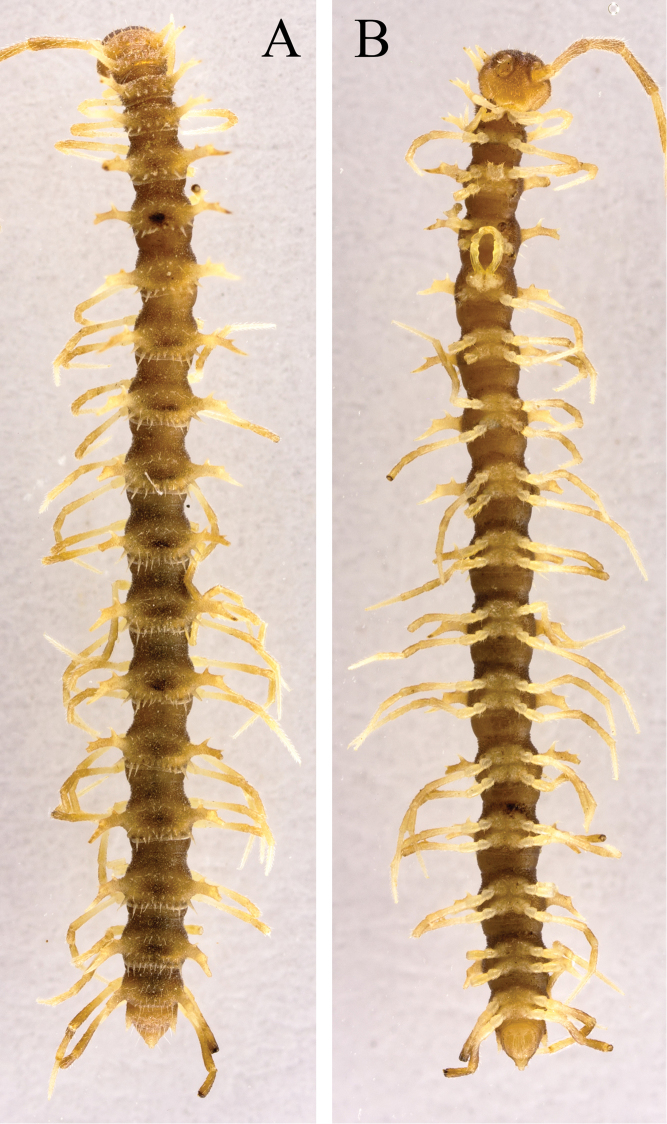
*Desmoxytes spiniterga* sp. n., ♂ paratype from near Cave Gui Dong II. **A–B** body, dorsal and ventral views, respectively.

Length *ca* 11 mm (holotype) or 12 mm (paratype), width of midbody pro- and metazonae 0.5 and 1.8 mm, respectively. In width, head < collum < segment 2–4 < 5–16. Coloration brownish to yellowish (Fig. 10). Antennae very long and slender, reaching back until posterior margin of segment 6.

**Figure 11.**
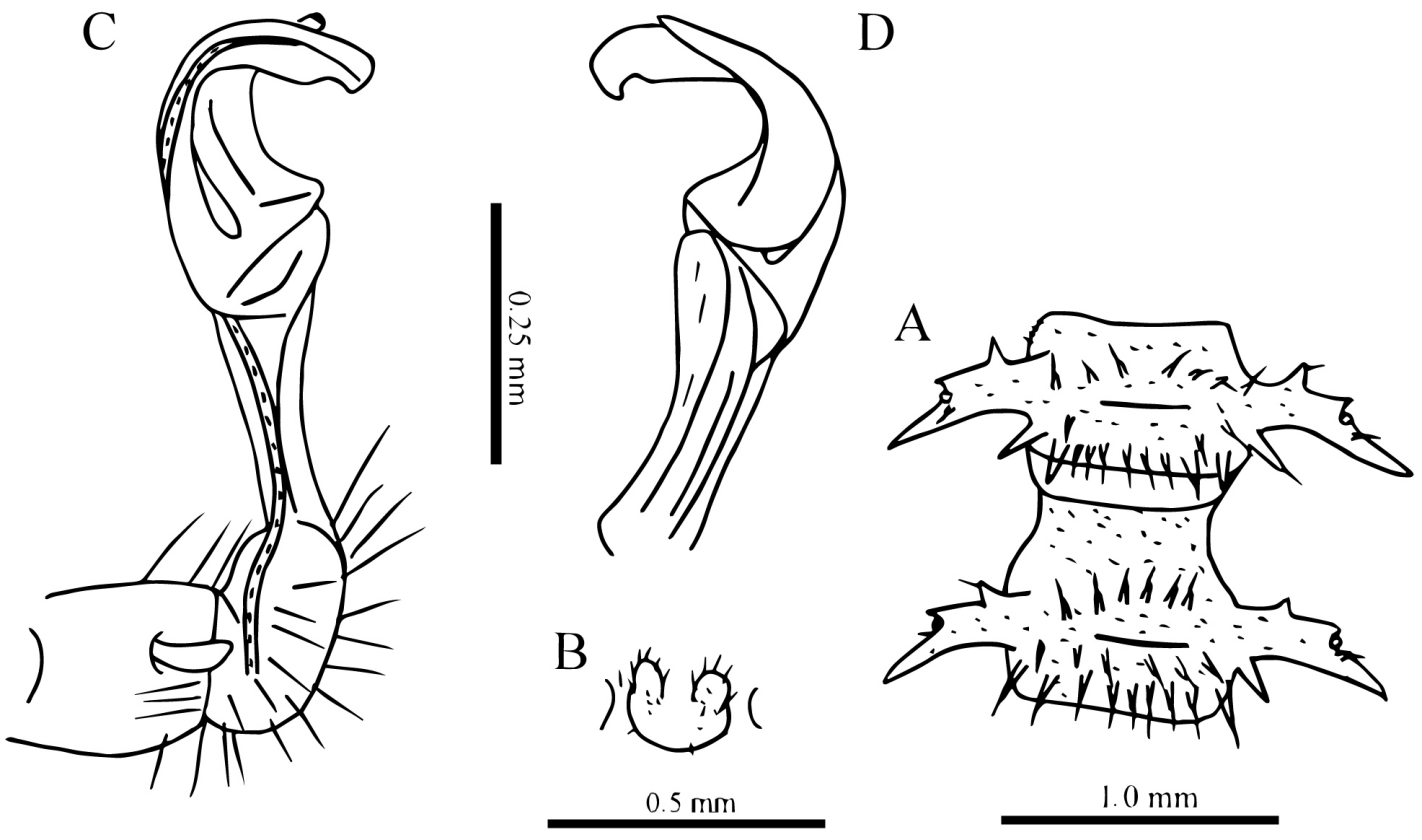
*Desmoxytes spiniterga* sp. n., ♂ paratype from near Cave Gui Dong II. **A** segments 9–10, dorsal view **B** sternal processes between coxae 4, ventral view **C–D** entire left gonopod and its distal half, mesal and lateral views, respectively.

Prozonae faintly microalveolate; metazonae rather microgranulate and shagreened. Collum (Fig. 10A) with 4+4(5) anterior, 2+2 intermediate and 2+2 posterior setigerous spines; following metaterga 2–8 with 3+3 anterior and 3(4)+3(4) posterior setigerous spines; in metaterga 9–18 increasingly more numerous, with a pattern of 3(4)+3(4) anterior, 2–4+2–4 middle (behind transverse sulcus) and 5(4)+5 posterior setigerous spines, in posterior row lateral one or two spines being much larger than others; metatergum 19 with the same spination pattern, but setigerous tubercles smaller and similar in size. Paraterga (Figs 10A, 11A) very strongly developed, antler-shaped, usually three-branched, paraterga on collum with two branches; paraterga 2–8 directed more dorsad than laterad; paraterga 9–18 directed laterad, but clearly ending up above dorsum, each with an additional small denticle at last incision; paraterga 19 directed caudad. Ozopores normal, lying at base of last incision of paraterga (Fig. 11A). Transverse sulcus present on segments 3–18, incomplete (Figs 10A, 11A).

Sterna moderately setose, cross-impressions shallow (Fig. 10B). A pair of paramedian processes between ♂ coxae 4 (Figs 10B, 11B). Legs without modifications, midbody legs *ca* 2.8 times (♂) as long as body height.

Gonopods (Figs 10B, 11C–D) with coxite about 1/3 as long as telopodite. Prefemur almost half as long as acropodite. Femorite short and slender, slightly enlarged distad. Solenophore clearly coiled and divided into a large spiniform lamella lateralis and a very distinct and coiled lamella medialis. Solenomere relatively long.

#### Remark.

Compared to cave-dwelling congeners, this species is much smaller and darker.

### Desmoxytes variabilis sp. n.

Taxon classificationAnimaliaPolydesmidaParadoxosomatidae

http://zoobank.org/F5F0F547-D6D8-4B91-8DF3-7988C32E1822

**Figure 12.**
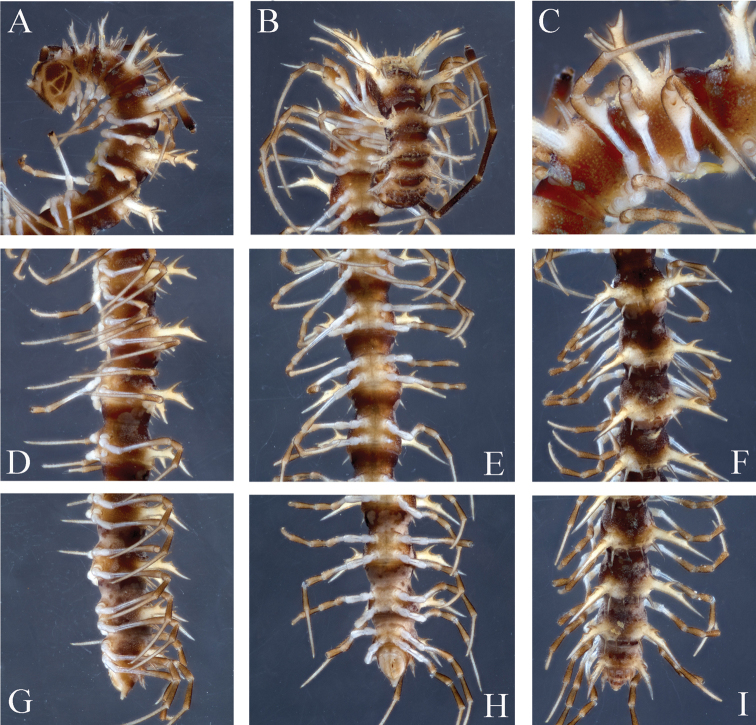
*Desmoxytes variabilis* sp. n., ♂ holotype from Cave Huoji Dong. **A–B** anterior part of body, lateral and ventral views, respectively **C** legs 5–7, ventral view **D–F** midbody segments, lateral, ventral and dorsal views, respectively **G–I** posterior part of body, lateral, ventral and dorsal views, respectively.

**Figure 13.**
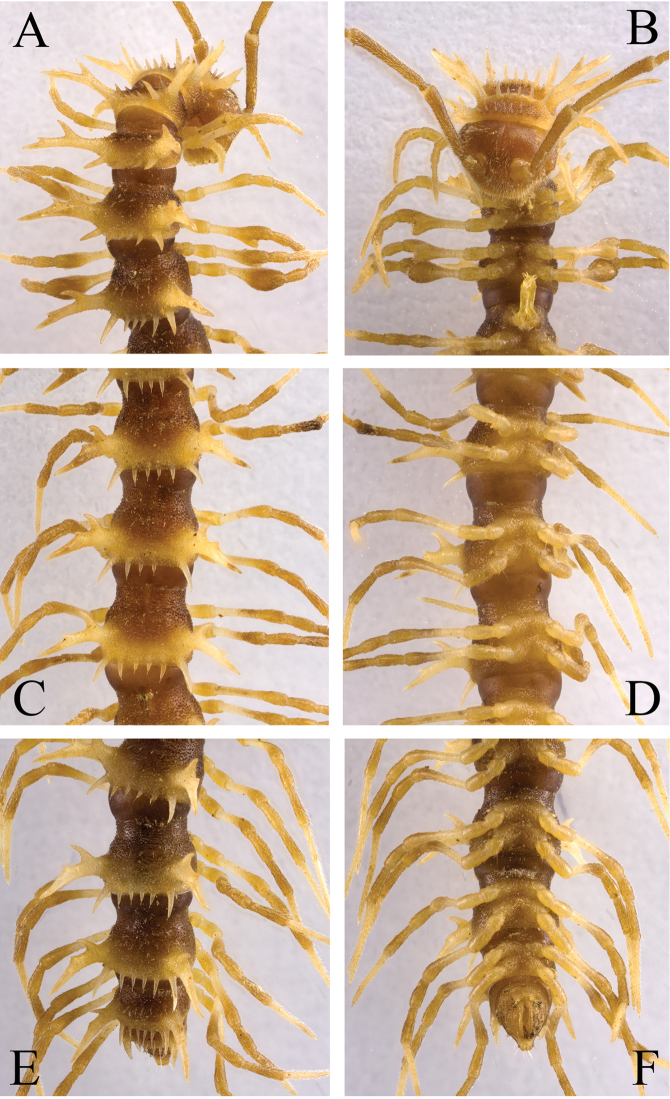
*Desmoxytes variabilis* sp. n., ♂ paratype from near Midong Cun. **A–B** anterior part of body, dorsal and ventral views, respectively **C–D** midbody segments, dorsal and ventral views, respectively **E–F** posterior part of body, dorsal and ventral views, respectively.

**Figure 14.**
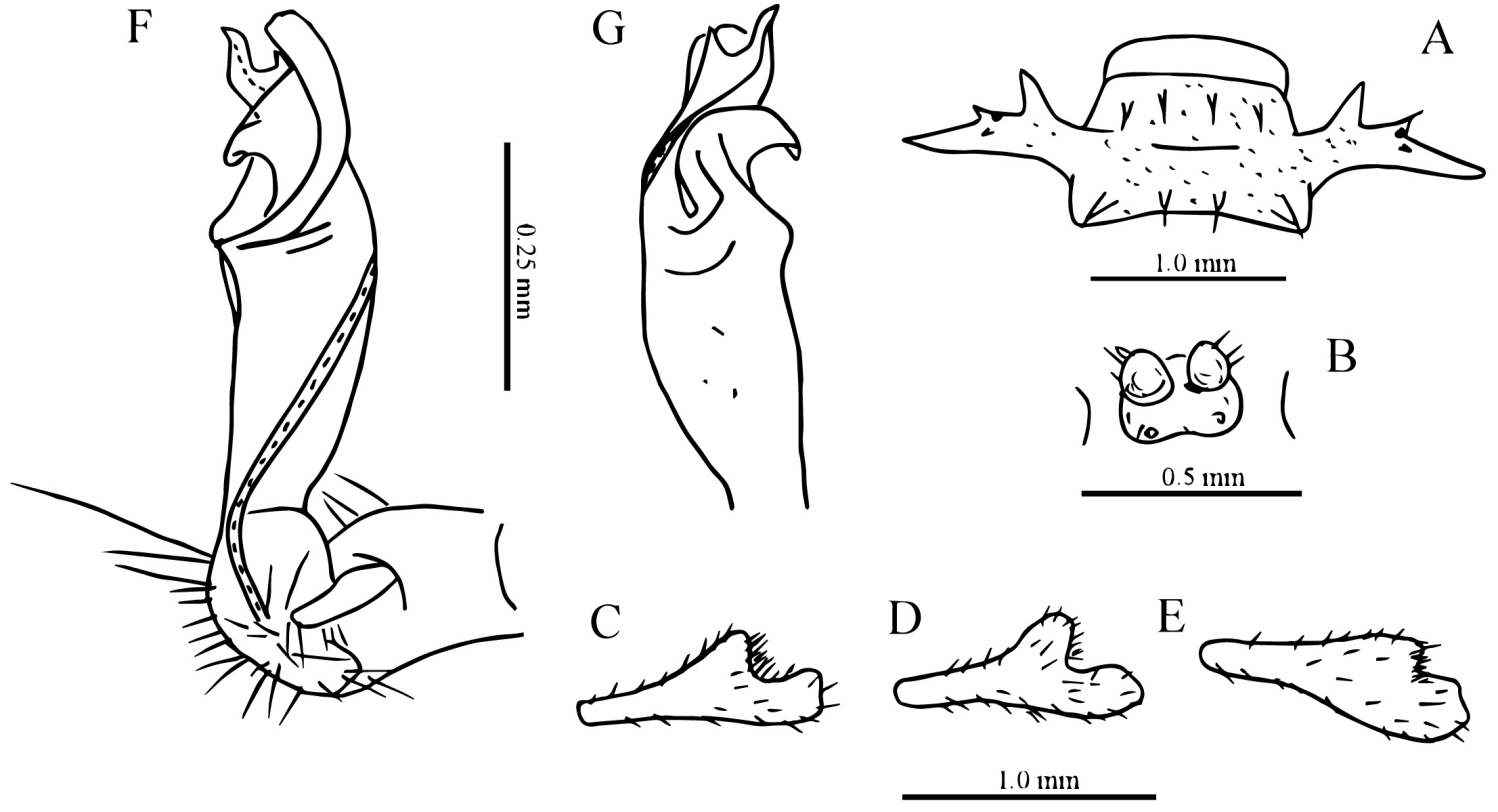
*Desmoxytes variabilis* sp. n., ♂ paratype from Cave Huoji Dong. **A** segment 10, dorsal view **B** sternal processes between coxae 4, ventral view **C–E** femora 5–7, front view **F–G** entire right gonopod and its distal half, mesal and lateral views, respectively.

Figs 1H , 12 , 13 , 14

#### Holotype

♂ (SCAU), China, Guangxi, Hechi, Fengshan Xian, Fengcheng Zhen, Cave Huoji Dong, 24°28'32"N, 107°03'28"E, *ca* 500 m a.s.l., 2015-VIII-3, leg. Jujian Chen, Xinhui Wang & Mingruo Tang.

#### Paratypes.

2 ♂, 3 ♀ (SCAU), same locality and collecting data as the holotype. 2 ♂, 1 ♀ (SCAU), 1 ♂, 1 ♀ (ZFMK), same County, Zhaiya Xiang, Laying Cun, Cave II Dong, 2015-VIII-4, leg. Jujian Chen, Xinhui Wang & Mingruo Tang. 3 ♂, 2 ♀ (SCAU), same County, Jinya Xiang, Hangdong Cun, Cave I Dong, 2014-VI-14, leg. Mingyi Tian, Weixin Liu, Haomin Yin & Xiaozhu Luo. 3 ♂, 2 ♀ (SCAU), Lingyun Xian, Sicheng Zhen, Wuzhishan, Cave Qianlongya, 2015-VI-10, leg. Mingyi Tian, Weixin Liu, Xinhui Wang & Mingruo Tang. 1 ♂, 1 ♀ (SCAU), Bama Xian, Yandong Xiang, Namen Cun, Cave Baiyan Dong, 2015-VIII-3; 3 ♂, 2 ♀ (SCAU), Tian’e Xian, Bala Xiang, Gandong Cun, Cave number VIII Dong, 2015-VIII-8. leg. Jujian Chen, Xinhui Wang & Mingruo Tang. 3 ♂, 3 ♀ (SCAU), China, Guangxi, Hechi, Huanjiang Xian, Mulun, way to Mashan Dong, 2007-V-21, leg. Louis Deharveng & Anne Bedos (CHIgx07-21-02). 2 ♂, 1 ♀ (SCAU), China, Guangxi, Hechi, Huanjiang Xian, near Midong Cun, 2007-V-23, leg. Louis Deharveng & Anne Bedos (CHIgx07-23-05).

#### Name.

To emphasize the metaterga showing a variable pattern of setigerous spines; adjective.

#### Diagnosis.

This species seems to be especially similar to *Desmoxytes nodulosa* Liu, Golovatch & Tian, 2014, from Cave II, Xiao’ao Xiang, Du’an Xian, Hechi, Guangxi, because both share very close patterns of metatergal ornamentation and particularly stout gonopods, but differs by the metaterga showing a variable pattern of setigerous spines, and the particularly complex gonopod solenophore.

#### Description.

All characters as in *Desmoxytes laticollis* sp. n., except as follows.

Length *ca* 17–21 (♂) or 20–24 mm (♀), width of midbody pro- and metazonae 1.0–1.2 and 2.8–3.8 (♂) or 1.5–1.8 and 3.0–4.0 mm (♀), respectively. Holotype 19 mm long, 1.0 and 2.8 mm wide on midbody pro- and metazonae, respectively. In width, head < collum < segment 2–4 < segment 5–16. Coloration (Figs 1H, 12, 14–15) varying from dark to light brownish; paraterga and posterior parts of metaterga finely yellow-brownish. Antennae long and slender, reaching back until posterior margin of segment 5 (♂) or segment 4 (♀) when stretched dorsally (Fig. 12A–B).

Prozonae faintly microalveolate; metazonae rather microgranulate and shagreened. Collum (Figs 12B, 13B) with 4+4 (or 5+5) anterior and 2+2 (or 3+3) posterior setigerous spines. Metaterga 2–18 (Figs 12–13) each with 2+2 (or 3+3) anterior and 2+2 (or 3+3) posterior setigerous spines, lateral spine of posterior row being much larger than others; metatergum 19 with 3+3 anterior and 2+2 posterior, similar, small, setigerous tubercles. Paraterga very strongly developed, antler-shaped, usually three-branched, each tip with an evident lateral seta (Figs 12–13). Paraterga of collum stout, two-branched; paraterga 2–5 directed more dorsad than laterad; paraterga 19 directed caudad, horizontal, lying level to dorsum; paraterga 6–18 directed obliquely upwards at *ca* 45°, in ♂ ending up clearly above dorsum (Figs 12–13), but paraterga slightly lower, shorter, subhorizontal and lying level to dorsum in ♀. Ozopores conspicuous, located at last incision of poriferous paraterga (Fig. 14A). Transverse sulcus obscure on collum and metaterga 2–4; more evident, but incomplete on metaterga 5–17 (Figs 12–13).

Sterna sparsely setose, cross-impressions very shallow (Figs 12–13). A paramedian pair of separated, short, rounded, poorly setose processes between ♂ coxae 4 (Fig. 14B). Legs long and slender, *ca* 2.5–2.8 (♂) or 2.0–2.2 (♀) times as long as body height; ♂ femora 5–7 each with a conspicuously densely pilose apophysis ventrally at about midlength (Figs 12C, 13A–B, 14C–E).

Gonopods (Fig. 14F–G) very short. Coxite less than 1/3 as long as telopodite. Prefemur less than half the length of acropodite. Femorite stout, apically with a distinct sulcus. Solenophore quite complex and compact, divided into two well differentiated lobes, a higher, bipartite and apically acuminate lamina medialis, plus a lower and curved lamina lateralis; solenomere short and flagelliform.

#### Remark.

This obviously troglophilic species is rather eurytopic, occurring both outside and inside caves. It shows a remarkably variable pattern of spination on collum and metaterga (Figs 12–13), but the gonopod structure remains stable.

### A key to *Desmoxytes* species currently known to occur in China

(modified after Golovatch et al. 2012; Liu et al. 2014)

**Table:** 

1	Paraterga spiniform (Figs 4, 6, 8)	**2**
–	Paraterga wing- (Fig. 2) or antler-shaped (Figs 10, 12–13)	**11**
2	♂ femora unmodified. Gonopod lamella medialis terminally evidently bifid	**3**
–	At least a pair of ♂ femora (6–7) humped ventrally	**4**
3	Lamella medialis devoid of a small midway lobe (Fig. 5B–C)	***Desmoxytes simplipoda* sp. n.**
–	Lamella medialis supplied with a small lobule at midlength (Fig. 7B–C)	***Desmoxytes similis* sp. n.**
4	At least ♂ femora 7 humped ventrally	**5**
–	Only ♂ femora 6 humped ventrally	**6**
5	Only ♂ femora 7 very evidently humped	***Desmoxytes longispina***
–	Both ♂ femora 6 and 7 evidently humped	***Desmoxytes spinissima***
6	Paraterga spiniform until segment 5, following paraterga very short, coni- to dentiform	***Desmoxytes lui***
–	Paraterga spiniform, very long, about as long as body height in ♂	**7**
7	Adult body relatively small, length < 20 mm, epigean	***Desmoxytes parvula***
–	Adult body much larger, length > 20 mm, mostly troglobites	**8**
8	Metaterga 2–19 not only with normally arranged setigerous tubercles, but also with a row of similar tubercles along posterior margin	**9**
–	Only metaterga 2–4 with several transverse rows of setigerous spines, following metaterga generally smooth, without tubercles along posterior margin	**10**
9	A pair of rounded setose processes between ♂ coxae 4 (Fig. 9A); gonopod lamella medialis with a distinct spine (Fig. 9D–E)	***Desmoxytes phasmoides* sp. n.**
–	A pair of square setose processes between ♂ coxae 4	***Desmoxytes minutubercula***
10	Gonopod postfemoral part sulcus evident, lamina lateralis with several small lobes; Guangxi	***Desmoxytes scutigeroides***
–	Gonopod postfemoral part without sulcus, lamina lateralis simple; Guizhou	***Desmoxytes getuhensis***
11	Paraterga wing-shaped	**12**
–	Paraterga antler-shaped	**15**
12	Collum the broadest segment (Figs 2A, 3A)	***Desmoxytes laticollis* sp. n.**
–	Collum narrower than midbody segments	**13**
13	♂ femora unmodified. Paraterga long and mostly subfalcate	***Desmoxytes eupterygota***
–	♂ femora 5–6 or 6–7 humped. Paraterga stout and suberect	**14**
14	Metaterga 2–19 with two transverse rows of setigerous spines. Gonopod telopodite suberect	***Desmoxytes planata***
–	Metaterga 9–19 with four transverse rows of setigerous spines. Gonopod telopodite subfalcate	***Desmoxytes scolopendroides***
15	♂ femora unmodified	***Desmoxytes spiniterga* sp. n.**
–	At least ♂ femora 6 humped	**16**
16	Only ♂ femora 6 humped	**17**
–	♂ femora 5–7 or 5–6 humped	**18**
17	A pair of tongue-shaped sternal processes between ♂ coxae 5. Gonopod femorite stout and curved	***Desmoxytes lingulata***
–	A pair of divergent sternal processes between ♂ coxae 5. Gonopod femorite elongated and suberect	***Desmoxytes cornutus***
18	Paraterga strongly and long branched, collum with 4+4 anterior and 1+1 posterior spines; Jiangxi	***Desmoxytes draco***
–	Paraterga usually three-branched, collum with at least 4+4 anterior and 2+2 posterior spines, sometimes even more numerous; Guangxi	**19**
19	Gonopod solenophore simple, solenomere separated at base from solenophore	***Desmoxytes nodulosa***
–	Gonopod solenophore complex (Fig. 14F–G), with several lobes, solenomere sheathed by solenophore	***Desmoxytes variabilis* sp. n.**

## Conclusion

The family Paradoxosomatidae (Polydesmida) is among the largest in the entire class Diplopoda (nearly 200 genera and >950 species, amounting to about 60% of the total species diversity in the Oriental fauna), but it is highly uncharacteristic of caves. Remarkably, *Desmoxytes* is the sole genus of Oriental paradoxosomatids that comprises numerous true cavernicoles (Golovatch 2015).

Now that *Desmoxytes* encompasses 41 described species, of which half derive from mainland China, a few observations seem to be noteworthy. Species group delimitation lies beyond the scope of the present paper, as it focuses only on the Chinese fauna. It also seems somewhat premature given the rapidly growing number of species described lately and certainly many more still to be found across China and Southeast Asia. However, superficially all *Desmoxytes* spp. that are presumed troglobionts are highly troglomorphic and have only been encountered in the karsts of southern China. Only among such congeners there are several that show remarkably long and spiniform paraterga, obviously an apomorphic troglomorphism.

The diversity of *Desmoxytes* as currently known may seem biased to cave-dwellers, in part because much of the collecting and taxonomic exploration efforts still focus on cavernicoles alone. Interestingly, however, in contrast to China, not a single troglomorphic species of *Desmoxytes* has been encountered in the numerous well-explored karsts of Thailand, Laos or Vietnam, even though epigean *Desmoxytes* are likewise very common and diverse in Indochina.

## Supplementary Material

XML Treatment for Desmoxytes laticollis

XML Treatment for Desmoxytes simplipoda

XML Treatment for Desmoxytes similis

XML Treatment for Desmoxytes phasmoides

XML Treatment for Desmoxytes spiniterga

XML Treatment for Desmoxytes variabilis
